# Decomposing and Modeling Acoustic Signals to Identify Machinery Defects in Industrial Soundscapes

**DOI:** 10.3390/s25164923

**Published:** 2025-08-09

**Authors:** Christof Pichler, Markus Neumayer, Bernhard Schweighofer, Christoph Feilmayr, Stefan Schuster, Hannes Wegleiter

**Affiliations:** 1 Christian Doppler Laboratory for Measurement Systems for Harsh Operating Conditions, Institute of Electrical Measurement and Sensor System, Graz University of Technology, Inffeldgasse 33, 8010 Graz, Austria; 2 voestalpine Stahl GmbH, voestalpine-Straße 3, 4020 Linz, Austria

**Keywords:** fault detection, signal decomposition, signal modeling, high noise, acoustic signals, signal processing, feature engineering

## Abstract

Acoustic sound-based condition monitoring (ASCM) systems, which typically utilize machine learning algorithms on established audio features, have demonstrated effectiveness under controlled conditions. However, their application in real-world industrial environments presents significant challenges due to complex and variable soundscapes with high noise and limited fault data. The presence of random interfering sounds and variability in operating conditions can lead to lower performance and high false-positive rates. To overcome these limitations, we propose a fault detection method that leverages the underlying physical characteristics of the sound signals. By investigating the components of the acoustic signal, we found that fault-related sounds can be modeled as exponentially decaying oscillations. This insight allows for the development of a physically based signal model, setting our approach apart from purely data-driven methods. Using this model, we developed a robust detection method based on a Generalized Likelihood Ratio Test (GLRT). The effectiveness of this approach was validated using both synthetic and real-world data from a steel industry facility. Our results demonstrate that the proposed model-based approach provides superior performance compared to standard audio features, particularly in high-noise conditions. On real-world data, the GLRT-based approach outperformed all audio features, as clearly shown by the Receiver Operating Characteristic (ROC) analysis. Specifically, the Partial Area Under the Curve (pAUC) of the GLRT is more than twice that of the best-performing audio feature, demonstrating good detection at significantly lower-false-positive rates compared to audio features. Furthermore, simulations showed that our method maintains robust detection down to a Signal-to-Noise Ratio (SNR) of −13 dB, significantly outperforming audio feature-based detection, which was limited to approximately −10 dB. The physically informed nature of our model not only provides a more reliable and robust solution but also enables the method to be generalized to other industrial scenarios with similar fault properties, offering broader applicability for reliable acoustic condition monitoring.

## 1. Introduction

In many industries, particularly those that rely on continuous manufacturing processes, the reliable performance of individual machines is of critical importance. Failures of machine components can lead to process interruptions or even complete production stoppages, resulting in substantial financial losses. This necessitates not only manual inspections but also automated condition monitoring systems [1].

Traditional condition monitoring approaches based on vibration sensors [2,3,4,5,6,7,8] are widely used but can be challenging to implement due to the higher costs and installation complexities associated with the required measurement equipment. As an alternative, acoustic sound-based condition monitoring (ASCM) has emerged as a promising approach [9]. The use of ASCM is supported by the fact that experienced maintenance personnel often identify early signs of damage through the acoustic signatures of machinery. Acoustic monitoring not only simplifies instrumentation by relying on a small number of sensors, specifically microphones, but also offers broad applicability across various machine types [1].

**Figure 1:**
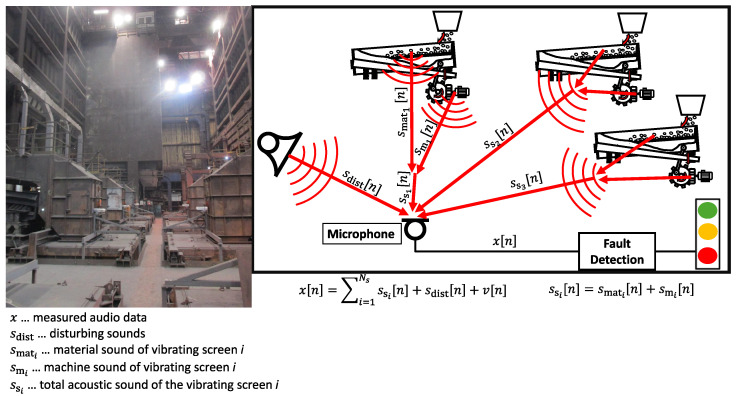
The picture on the left shows the inside of the burden preparation area of an iron-making blast furnace. On the right is a schematic illustration of the signal composition from several acoustic sources within the facility.

However, microphones capture not only the sounds emitted by the target machine but also the acoustic environment of industrial facilities, including background noise generated by other machines and human activities. In this work, we refer to the signal-to-noise ratio (SNR) as the ratio between the machine signal and the background sound, which is typically lower in microphone measurements compared to vibration-based measurements. This issue becomes even more pronounced in environments where multiple machines operate simultaneously, as is common in industrial settings. This situation is illustrated in Figure 1, which shows both the industrial environment and the resulting mixture of acoustic sources for the addressed application.

Previous research has developed a broad spectrum of methods for monitoring individual machines or their components. Many of these methods employ machine learning techniques that leverage the acoustic features of faults. For example, some studies have focused on diagnosing faults in specific machinery, such as gearboxes [10], industrial fans [11], or milling tools [12,13]. Other works have reviewed the application of acoustic analysis for condition monitoring of specific components like induction motors [14] and roller bearings in railway networks [15]. Deep learning models, in particular, have been used for tasks like predicting remaining useful life and classifying faults in components and engines [16]. However, these existing approaches have two key limitations that are not appropriate for our application. First, they primarily focus on fault detection in single machines or their components [10,11,12,13,14]. This limits their applicability in complex industrial settings because the methods struggle to generalize from clean, single-source environments to real-world scenarios with multiple overlapping sound sources, machine variability, and high levels of background noise. Second, these data-driven methods typically require extensive datasets containing examples of faults to train their models effectively [14,16], which is often impractical, since these approaches often do not generalize well beyond the specific machines or components they are trained on. In dynamic, multi-machine environments like those found in heavy industry, the need for manual labeling or retraining across different machines severely limits their scalability and practical use.

An alternative strategy involves the use of anomalous sound detection (ASD) systems. These techniques have proven effective for detecting anomalous sounds in a variety of contexts [17,18,19,20], primarily because they can operate using only normal operating data through outlier detection. Nevertheless, ASD approaches may also identify outliers caused by unrelated or unknown events, such as maintenance activities or signal horns, which are undesirable in the context of fault detection [21].

To address these challenges, an alternative approach is to explicitly detect faults by leveraging the underlying physical characteristics of fault signals. This requires a comprehensive understanding of all sound sources and their contributions to the overall acoustic signal. In this work, we aim to characterize the physical properties of these signals and develop a mathematical model to describe them. By analyzing the individual components using suitable signal analysis tools, we can construct a signal model that enables alternative detection strategies. In addition, such a model facilitates the generation of artificial fault data, which is essential for evaluating and comparing different detection methods in the absence of sufficient real fault data.

While concepts such as modeling exponentially decaying oscillations and employing general likelihood ratio test (GLRT)-based detection are well established in fields like radar signal processing [22], structural health monitoring [4], and vibration-based condition monitoring [6], their direct application to acoustic condition monitoring in complex industrial environments remains largely unexplored. In this work, we therefore do not claim novelty in the individual methods themselves, but rather in their adaptation and integration into a physically motivated framework specifically tailored to challenging industrial soundscapes. This approach can also be widely applied to industrial environments, particularly those involving vibrating or rotating machinery.

The main contributions of this work are as follows. We develop a physically based signal model that captures the characteristic properties of machine, material, and fault sounds in complex industrial environments. We then demonstrate how this model can be applied to achieve robust fault detection by comparing its performance with standard audio feature-based approaches. Finally, we leverage the model to generate realistic synthetic data, addressing the common challenge of limited fault examples needed for comprehensive testing and validation. These physically grounded methods address key limitations of existing data-driven approaches, particularly their dependency on extensive labeled datasets and their narrow applicability to isolated machine components.

Building on this, we present our approach for monitoring large machinery within the acoustically complex environment of a burden pre-processing facility. To tackle this challenge, we have developed a microphone array for data acquisition that enables monitoring of multiple machines within a defined surveillance area. In Section 2, we describe the sound acquisition system, the recorded acoustic signals, and the test setups for recreated fault scenarios in detail. A signal model based on physical principles and signal characteristics is then presented in Section 3 and Section 4. Finally, Section 5 compares the detection capabilities of the model-based detector with those of conventional audio features, demonstrating how the signal model approach provides a more comprehensive and informative signal description, thereby enhancing fault detection.

## 2. Measurement Set-Up and Characteristics of Signals from an Industrial Facility

When developing the microphone array for data recording, the difficult environmental conditions in a burden pre-processing facility must be taken into account. The sound level in the hall was found to be consistently high, averaging around 95 ${\mathrm{dB}}_{\mathrm{SPL}}$. Additionally, the hall is subjected to high dust loads and seasonal temperature fluctuations, which necessitated the use of a dust-protected set-up and microphones with a high maximum sound pressure level.

### 2.1. Measurement Set-Up

**Figure 2:**
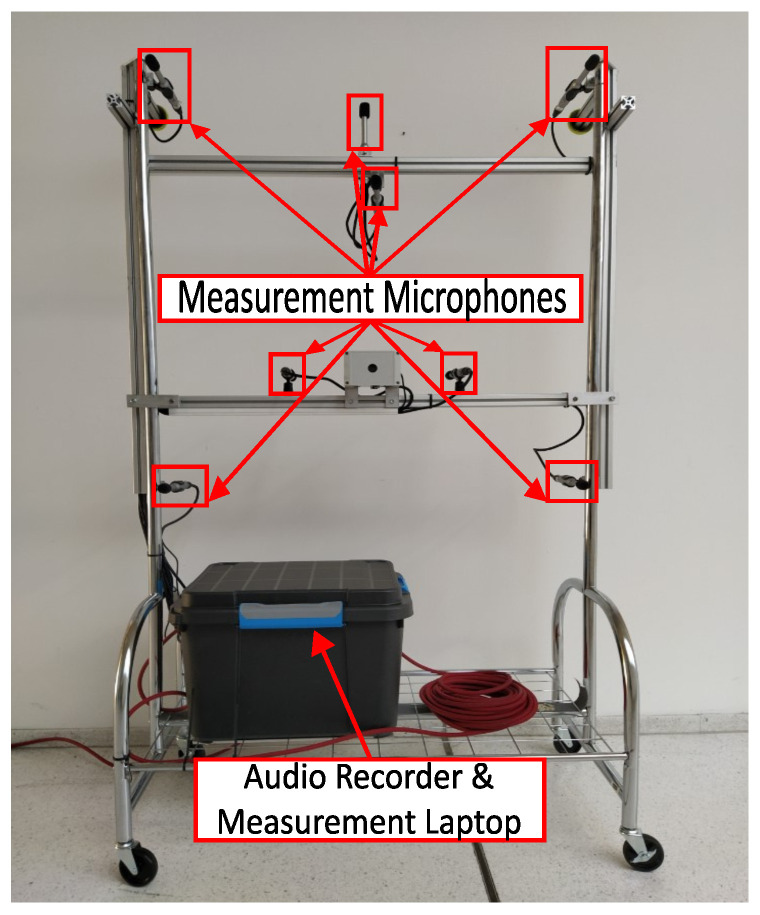
Image of the measurement set-up. The measurement set-up consists of a microphone array with eight measurement microphones and a dust-protected box for the audio recorder and the measurement laptop.

An array of 8 ECM8000 measurement condenser microphones from Behringer, Willich, Germany was deployed for the microphone array setup. These microphones were selected because of their flat frequency response spanning from 20 to 20,000 Hz, making them particularly suitable for this application. The audio signals were captured using an 8-channel Zoom F8n MultiTrack field recorder at a sampling rate of 48 kHz. To enable autonomous operation, the system was controlled via a measurement laptop equipped with remote access capabilities. The same system was also used in [20]. The specific arrangement and orientation of the microphones are illustrated in Figure 2. Foam covers were fitted over the microphones to shield them from the considerable dust levels in the hall. Additionally, other sensitive components such as the audio recorder and the measurement laptop were placed inside a dust-protected enclosure to ensure their protection.

To allow for flexibility in the data acquisition process, the measurement duration and number of channels can be adjusted in the software to meet the specific requirements of each experiment. The resulting audio data are saved in the .wav file format.

### 2.2. Audio Measurements

**Figure 3:**
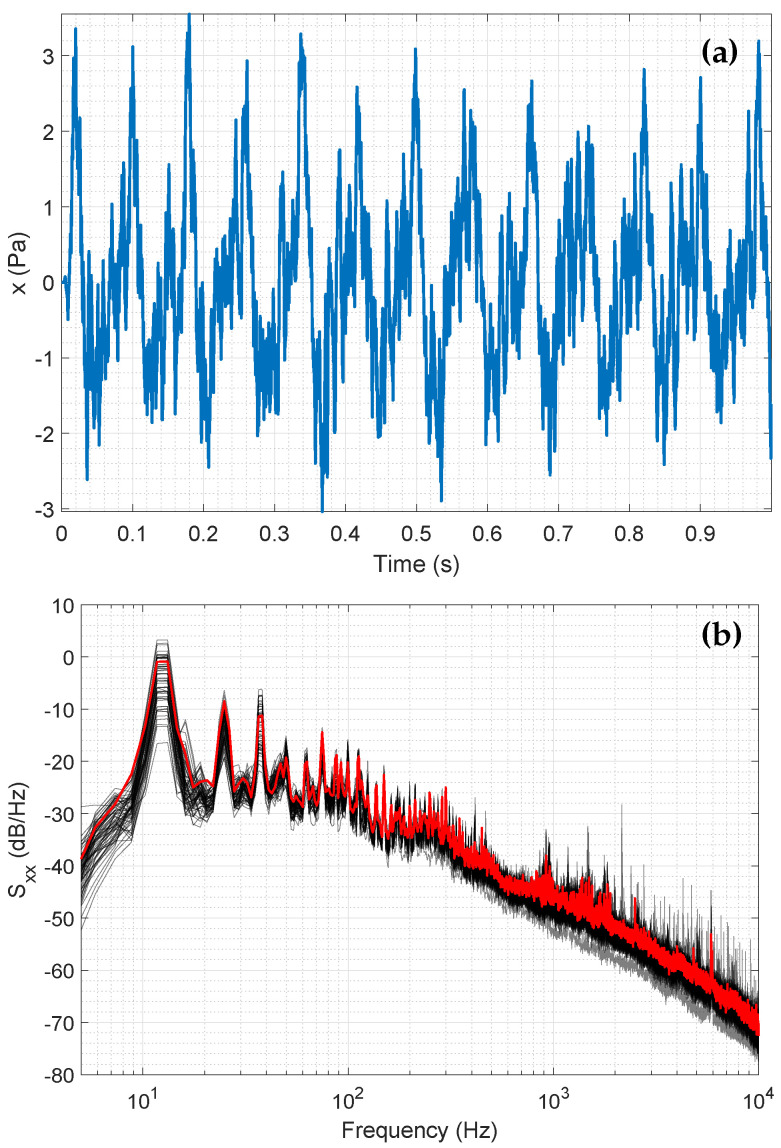
(**a**) Time series of a generic audio measurement. A low-frequency harmonic with additional higher-frequency components can be determined in the time-domain plot. (**b**) Power spectral densities of different measurements in the hall. Different operating states of the machinery in the hall span a signal space for the normal operating states. A generic signal curve is shown in red.

To obtain sound pressure measurements from the digital audio data, we utilized the specifications of the microphones and the recording device. The resulting one-second-long snippet of the time series of an example measurement in the burden pre-processing facility is shown in Figure 3a, where a low-frequency harmonic with additional higher-frequency components is evident. In Section 3 and Section 4, the properties and physical backgrounds of these components are discussed in more detail.

The acoustic signal in the industrial facility is a superposition of different sound sources. Raw materials are pre-processed in these facilities using industrial screens. Multiple screens operate simultaneously to increase throughput capacity. These screens can either be switched on or off depending on the material requirements of the blast furnace. This results in a large number of operating state combinations, each occupying a different state in the frequency domain. Thus, the normal operating states span a certain signal space. A previous study [23] demonstrated that the acquired acoustic signals belong to the category of short-time stationary signals. As a result, the power spectral density (PSD) analysis is an appropriate method to investigate the frequency characteristics of the data. The normal operating states exhibit distinct PSDs, as shown in Figure 3b. The PSDs vary in level across the different operating states, indicating differences in the acoustic sound scene.

As in the time domain, the low-frequency components can also be observed in the PSDs of the various normal operating states in each measurement.

### 2.3. Recreation of Fault Case

**Figure 4:**
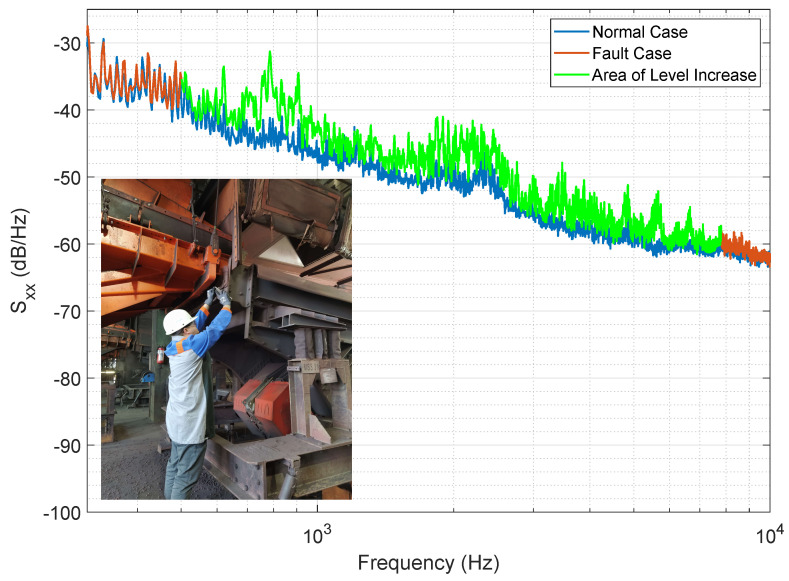
PSDs for the normal condition and the recreated fault case. The fault case manifests itself in a level increase in a specific frequency band (green line). The picture in the left lower corner of the plot shows the recreation experiment of the fault case.

To analyze the audio signal of fault cases in more detail and further be able to develop a signal model, it is essential to have audio data of the fault cases too. Since these data are not readily available, we recreated fault events on the machines and recorded the sounds. In most fault cases, metallic parts break, such as a spring rupture, and vibration causes these parts to hit each other, producing a distinctive metallic knocking sound. This knocking sound is a typical fault indicator in vibrating screens. We reproduced several real fault cases based on this physical understanding. Experienced maintenance personnel helped during the experiments to ensure the sounds closely matched real fault cases. In this way, we recreated the mechanical fault as accurately as possible by mechanically reproducing the same conditions. Figure 4 illustrates the experimental set-up, and the figure also displays the PSDs of the normal operating condition just before the fault experiment and during the recreated fault.

This approach enabled us to record multiple datasets with recreated but authentic-sounding fault cases. The experiments were conducted on various machines within the facility, and the data acquisition system was fixed in position to maintain consistency in the measurements. Furthermore, we conducted these measurements during normal facility operation, ensuring a realistic background sound environment.

## 3. Signal Modeling

The audio signal in a pre-processing facility is a complex mixture of several sound sources. The most important contributions to the overall signal include the sounds generated by the sieving and conveying process of the raw materials, the sounds of the several-meter-long vibrating screens that are switched on, and various other sound sources in the facility. In order to gain a better understanding of the individual signal components and to identify possible faults, we will subsequently propose a signal model for the sound scene in the facility.

**Figure 5:**
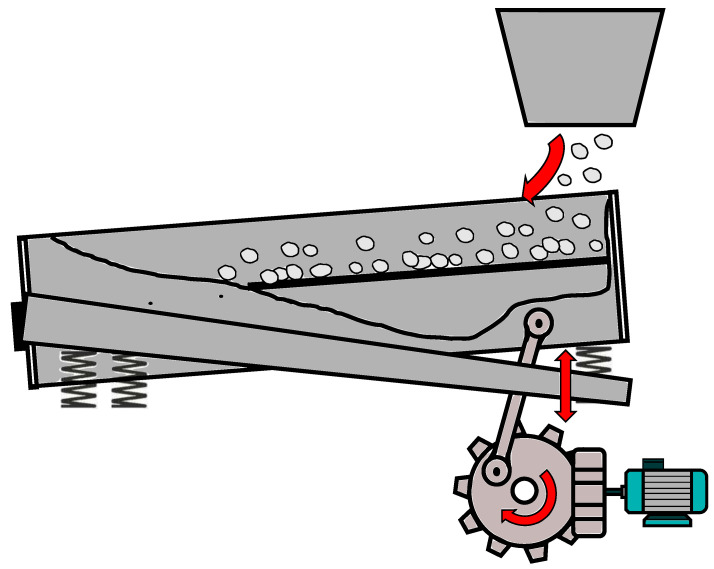
Schematic drawing of a vibrating screen. An asynchronous motor excites the screen to vibrate via a gear system. The raw material falls down from the so-called bunker and is screened and conveyed along the wacker plate.

Figure 5 depicts a schematic drawing of a single vibrating screen used for conveying and sieving raw materials. Material is fed from a bunker above onto the wacker plate of the vibrating screen. During operation, an asynchronous motor drives a gearbox that excites the wacker plate into vibration. Mounted on a spring system, the plate conveys and screens the material through its vibrating movement. The resulting audio signal consists of both the sound generated by the vibrating machine and the noise produced by the conveyed and screened material.

Based on these considerations, we can establish a signal model for the acoustic sound of the *i*-th screen as

(1) $$s_{s_{i}}\left[n\right]=s_{m_{i}}\left[n\right]+s_{{\mathrm{mat}}_{i}}\left[n\right],$$

with n=0,1,…,N−1 being the sampling index, *N* being the number of samples, $s_{m_{i}}$ being the machine sound, and $s_{{\mathrm{mat}}_{i}}$ being the material sound.

Given that $N_{s}$ screens operate simultaneously, we can express the overall acoustic signal $s_{s}$ as the sum of all acoustic sound models for the individual active screens and obtain

(2) $$s_{s}\left[n\right]=\sum_{i=1}^{N_{s}}s_{s_{i}}\left[n\right]=\sum_{i=1}^{N_{s}}s_{m_{i}}\left[n\right]+s_{{\mathrm{mat}}_{i}}\left[n\right].$$

As shown in Figure 1, the measurement signal *x* is represented as the sum of the acoustic signal from the screens $s_{s}$, additional disturbing sounds $s_{\mathrm{dist}}$, and measurement noise *v*. By including an additional fault sound $s_{\mathrm{fault}}$, the complete model for the measurement signal *x* is defined as

(3) $$x\left[n\right]=s_{s}\left[n\right]+s_{\mathrm{dist}}\left[n\right]+s_{\mathrm{fault}}\left[n\right]+v\left[n\right].$$

Using Equation (2) and given that the running screens are excited at the same frequency, the individual machine signals $s_{m_{i}}$ can be aggregated into a single overall machine component $s_{m}=\sum_{i=1}^{N_{s}}s_{m_{i}}$. Similarly, the individual material signals $s_{{\mathrm{mat}}_{i}}$ can be combined into a single overall material component $s_{\mathrm{mat}}=\sum_{i=1}^{N_{s}}s_{{\mathrm{mat}}_{i}}$, due to the inherent randomness of the material sound from each screen. Consequently, the measurement signal can be expressed as

(4) $$x\left[n\right]=s_{m}\left[n\right]+s_{\mathrm{mat}}\left[n\right]+s_{\mathrm{dist}}\left[n\right]+s_{\mathrm{fault}}\left[n\right]+v\left[n\right]$$

Given the high sound level in the facility (around 95 ${\mathrm{dB}}_{\mathrm{SPL}}$), the measurement noise *v* can be considered negligible. In the subsequent subsections, we will employ established signal analysis techniques to examine each of the other signal components in greater depth.

### 3.1. Disturbing Sound $s_{\mathrm{dist}}$

Disturbing sounds are background sounds, e.g., from signal horns that are installed in the hall for safety reasons. There are different types of signal horns, including those that emit chirping signals in different frequency ranges. These signals are all non-stationary, i.e., their statistical characteristics change over time and it is possible to detect them with a stationarity assessment. In addition, workers can cause disturbing sounds by cleaning the machines with compressed air, generating random, broadband, non-stationary signals. Other worker activities can also contribute to non-stationary signals. Other processes during screen operation can generate disturbing sounds. In some cases, conveyed particles can hit the walls of the screen and trigger oscillations that generate random, isolated knocking sounds. The data containing disturbing sounds are not used to evaluate the signal components, as their inclusion would unnecessarily complicate the analysis. However, we use such data to evaluate the detection performance of different detection approaches as they better represent real-world conditions and allow us to test the robustness of the approaches in practical scenarios. Therefore, we can neglect $s_{\mathrm{dist}}$ for the following evaluation.

### 3.2. Machine Signal $s_{m}$

**Figure 6:**
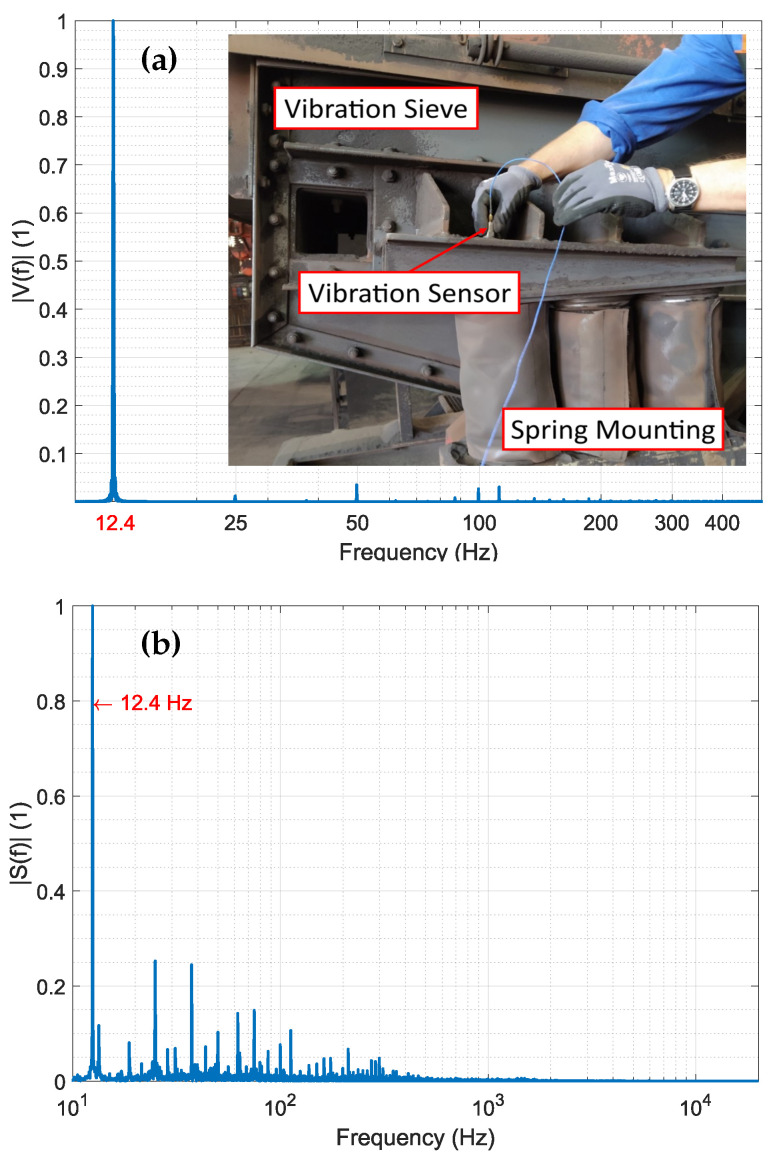
(**a**) Normalized spectrum of vibration measurements. The vibration of the screen is excited with an asynchronous motor via a gear box. To determine the vibration spectrum of the screen, a piezo-sensor was used. The fundamental frequency is marked in red. (**b**) Normalized spectrum of audio measurements. The main peaks in the audio spectrum are the fundamental frequency at 12.4 Hz and higher harmonics. Thus, the screen vibration is a main component of the audio signal.

To support our thesis that the low-frequency component of the audio signal is caused by the vibration of the screen itself, we conducted vibration measurements on the machines using a vibration sensor. A periodic component in the audio signal is expected due to the periodic excitation of the screen vibration. The normalized spectrum of the vibration measurements is shown in Figure 6a, along with a photo of the measurement set-up.

From the vibration measurements, we have determined that the fundamental frequency of the machine is 12.4 Hz and that higher harmonics can also be observed in the spectrum. As shown in Figure 6b, these frequencies are also present in the audio spectrum of the recorded signal. This implies that the machine vibration indeed generates an audio signal with a fundamental frequency outside the audible range. In our proposed signal model, this component, which is composed of the fundamental and its higher harmonic, is called the machine signal $s_{m}$.

Due to the low-frequency range outside the audible range, we suggest removing the machine-related signal component $s_{m}$ using high-pass (HP) filtering. As shown in Section 3.3 and Section 4, the other signal components mainly contribute to the overall signal in the higher-frequency range. Therefore, HP filtering is a legitimate method for removing the machine-related signal component $s_{m}$.

An important parameter in the HP filtering process is the cut-off frequency. After evaluating the spectra of various audio signals from the pre-production facility, it was concluded that the higher harmonics resulting from the vibration of the screen have a significant influence up to approximately 300 Hz. Furthermore, it is shown in Section 4 that the fault case occurs at frequencies above 500 Hz. Therefore, the cut-off frequency was defined as 300 Hz for the purpose of extracting mainly the signal component caused by the machine vibration. Although this signal has relatively high amplitudes, it is acoustically weakly perceptible. This can be explained by the low-frequency components of the signal, which are poorly perceived by the human ear.

**Figure 7:**
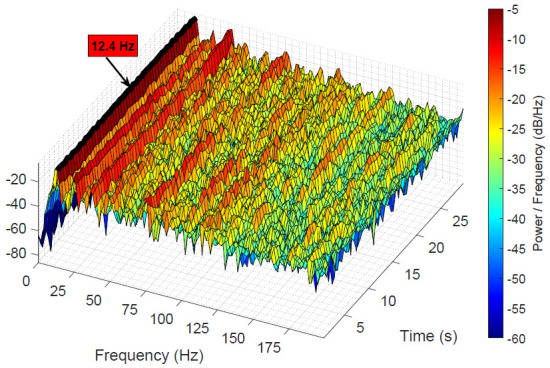
STFT of the recorded facility audio data in the low-frequency regime. It shows the change in the amplitudes of the higher harmonics.

Alternative methods for extracting the machine signal $s_{m}$, such as time-synchronous averaging (TSA), are not very suitable due to their dependence on the signal being constant over an extended period of time. As shown in Figure 7, the machine signal undergoes rapid changes in the facility. This short-time-frequency transform (STFT) clearly indicates that the fundamental frequency’s amplitude remains relatively constant, while the higher harmonic amplitudes change quickly, accounting for the time signal’s fluctuations.

It is important to note that even after removing the machine signal component $s_{m}$ from the measurement signal *x* by means of HP filtering, the fault signal is still audible. As discussed in Section 4, the fault signal $s_{\mathrm{fault}}$ is mainly present in a higher-frequency range, supporting the proposed signal decomposition.

### 3.3. Material Sound $s_{\mathrm{mat}}$

Considering the signal model, when we subtract the machine signal $s_{m}$ from the measurement signal *x*, we obtain two signal components: the conveying material signal $s_{\mathrm{mat}}$ and the possible fault signal $s_{\mathrm{fault}}$. Therefore, if the machine is functioning normally (without any fault), this method allows us to access the signal component caused by the material conveyance.

In the course of this process, the sieving material parts are excited by the vibration of the screen. As a result, the material rattles over the wacker plate and is conveyed along it. The conveying process involves the collision of material particles with parts of the screen, as well as their interaction with each other. These events give rise to the sound that we refer to in this paper as material sound $s_{\mathrm{mat}}$. Due to the stochastic nature of these collisions, this signal component can be considered as a random signal.

**Figure 8:**
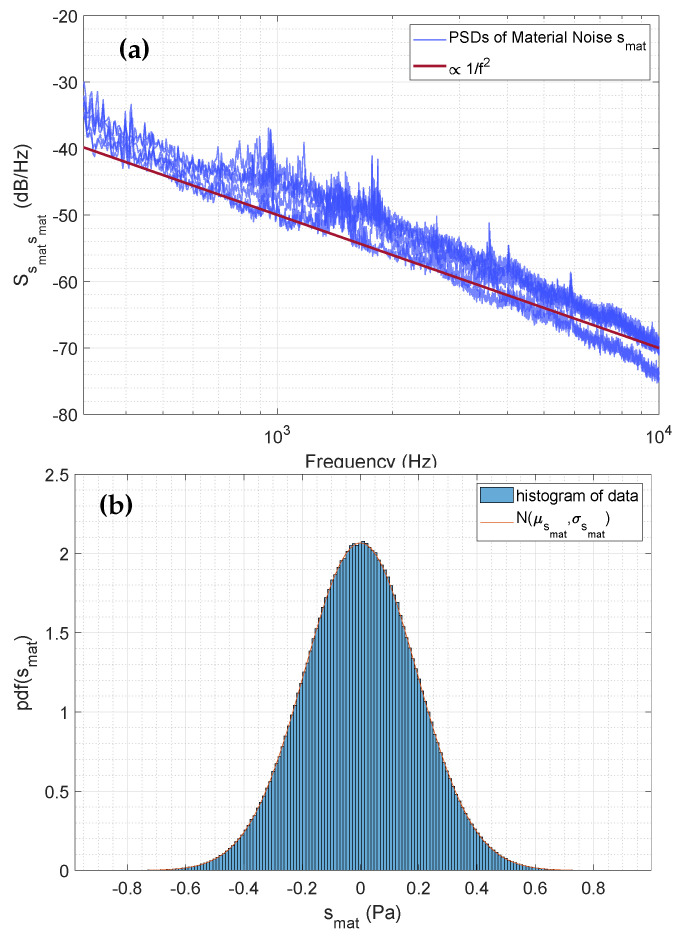
(**a**) PSD of the material signal $s_{\mathrm{mat}}$. The trend of the PSD shows a behavior proportional to $1/f^{2}$. Thus, the material signal $s_{\mathrm{mat}}$ can be described as brown (random walk) noise. (**b**) Histogram of the material sound signal values. The histogram shows a Gaussian distribution of the signal values of the signal component $s_{\mathrm{mat}}$.

The PSD analysis of $s_{\mathrm{mat}}$ reveals that the signal follows a $1/f^{2}$ behavior within a specific frequency range (see Figure 8a). Thus, the material sound can be characterized as brown noise (random walk noise) behavior in the frequency range up to 10 kHz. The PSD also exhibits a steeper negative slope at around 10 kHz. However, as we will demonstrate later, the high-frequency component of the material sound (above 10 kHz) is not significant for damage detection.

The material sound values follow a Gaussian distribution, as shown in Figure 8b. Since brown noise is correlated Gaussian noise, we performed a correlation analysis to determine the temporal correlation length of the signal. The analysis revealed that data with a length of approximately 15 ms are correlated. Therefore, we can describe this signal component as a correlated Gaussian noise process.

These findings enable us to establish a mathematical model for the random process as

(5) $$p\left(s_{\mathrm{mat}}|\Sigma ,I\right)=\frac{1}{\sqrt{det(2\pi \Sigma )}}\;exp\left(-\frac{s_{\mathrm{mat}}^{T}\Sigma^{-1}s_{\mathrm{mat}}}{2}\right),$$

with Σ being the covariance matrix of the random process.

In the healthy state of the facility, the signal space is spanned by the sum of the machine sound and the material sound. In the following section, we will investigate a fault sound and develop a signal model based on its characteristics.

## 4. Fault Sound $s_{\mathrm{fault}}$

The machine faults were recreated and recorded as described in Section 2.3. PSD analysis of these fault signals reveals a noticeable increase in the signal level within a certain frequency range. However, this increase is very small, ranging from 0.5 dB to 2.3 dB on average, resulting in a low SNR of −10 dB to −1.5 dB. Nevertheless, the fault signal is audible in the audio signal.

### 4.1. Signal Model Proposal

**Figure 9:**
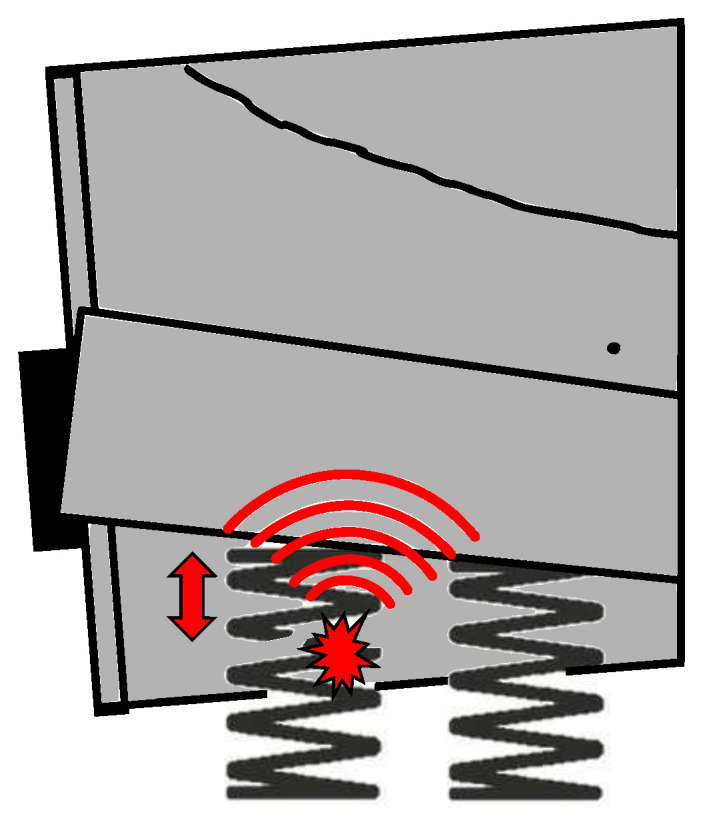
Schematic drawing of a spring rupture of a vibrating screen. Most machine damages are related to the springs. Even though the spring is ruptured the screen continues to vibrate. Therefore, metallic parts (spring parts) strike the machine with the vibration frequency. This causes an additional sound source and can be heard in the audio data.

Most cases of damage in the machine occur in the spring bearing. Under a continuous load, the spring may break, causing repeated striking against other metal parts due to the vibrations of the screen. This behavior is depicted schematically in Figure 9 and can be heard in the audio signals as a knocking sound. The impact of broken spring components on other metal parts of the screen is excited by the fundamental frequency of the screen vibration. Therefore, the signal model proposed for the fault case $s_{\mathrm{fault}}\left[n\right]$ is a knocking sound p[n] convolved with a pulse train *i* and can be written as

(6) $$s_{\mathrm{fault}}\left[n\right]=p\left[n\right]*i\left[n\right]\;\cdot \;u\left[n\right]$$

with u[n] being the unit function to restrict the function to the positive range. The impulse train i[n] is

(7) $$i\left[n\right]=\sum_{m=-\infty }^{\infty }\delta \left(n-mT_{m}\right).$$

Due to the excitation of the pulses via the vibration of the screen, $T_{m}$ represents the period of vibration of the screen.

**Figure 10:**
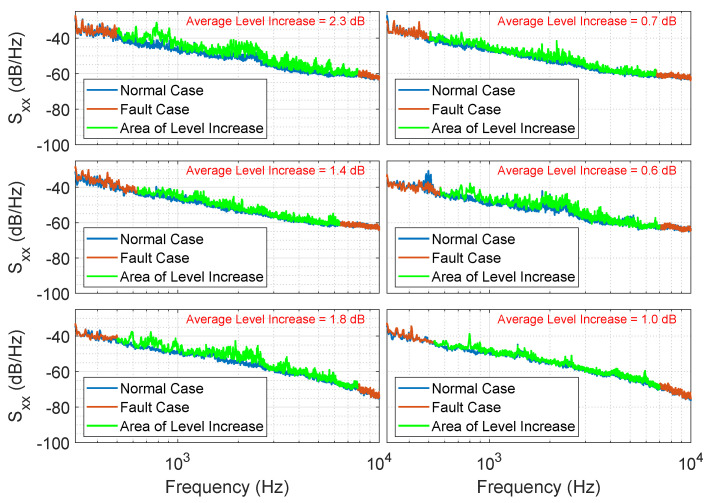
PSDs of some example audio signals x in normal conditions and in fault conditions. The level in the PSD is raised in a certain frequency range in the event of a fault. This means that the fault case is noticeable as a broadband signal component.

PSD analysis (Figure 10) revealed that the fault condition only affects a specific frequency band. The frequency response of a fault case is not a straight line, indicating the presence of frequency components with higher signal power.

Thus, the fault case presents itself as a band-limited signal within a specific frequency range. Additionally, the signal is expected to decay exponentially over time as it is excited in a pulse-like manner. In order to establish a general and physically grounded model for this signal component, we aim to describe its generation using a physical approach. Mechanically speaking, in the event of a fault, loose metal parts impact the side wall of the machine, which in turn excites the vibration modes of the screen. Due to the complex geometry of the screen, this impact leads to the excitation of multiple vibration modes, resulting in the emission of an acoustic fault signal p[n]. A physical description of such a signal can thus be achieved by representing the knocking sound as a sum of damped sinusoidal signals with different frequencies, amplitudes, phases, and exponential decay. Thus, p[n] can be written as

(8) $$p\left[n\right]=\sum_{k=1}^{N_{f}}A_{k}cos\left(2\pi f_{k}n+\phi_{k}\right)\;\cdot \;exp\left\{-\frac{n}{\tau_{k}}\right\}.$$

The exact number of different frequencies $N_{f}$ in the physical model is not yet known. In the PSDs for the fault case, it can be seen that the amplitudes $A_{k}$ decrease with increasing normalized frequencies $0\leq f_{k}<1/2$. In order to take into account the phase shifts of the oscillation modes, it is necessary to use the phase parameters $\phi_{k}$. $\tau_{k}$ represents the decaying constants that can change for different frequencies. With this knowledge, it is possible to generate synthetic data that acoustically matches the measured fault case. In practice, we have chosen $N_{f}=60$ as a first approximation for the generation of synthetic signals, as increasing the number of frequencies did not significantly improve the acoustic similarity of the generated signal.

**Figure 11:**
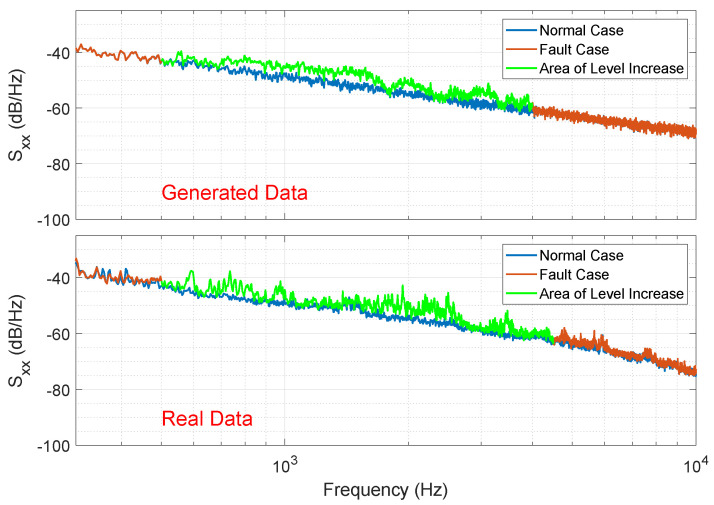
The upper plot shows the PSD of the generated synthetic fault signal with a sum of decaying sinusoidal signals as the knocking sound p[n]. The lower plot shows the PSD of one dataset of the recorded simulated fault case.

The measured fault case data reveals that the individual knock sounds exhibit variations. This implies that not every knock pulse has the same characteristics. To account for this, we further incorporated slight variations in the amplitudes, frequencies, and phases of the sinusoidal signals from one knock pulse to another when generating the synthetic data. The frequency response of the resulting synthetic fault signal generated using this model closely resembles that of an emulated fault case, as shown in Figure 11.

While this approach enables us to generate data that exhibits similarities in the sound and frequency behavior to the experimental data, subjective assessments of sound quality are not a reliable measure for evaluating the validity of the model. Hence, we need to employ additional methods to validate the model’s accuracy.

### 4.2. Signal Model Verification

To validate our assumption that the fault signal can be modeled as a sum of exponentially decaying sinusoidal signals, we used an matched filter bank (MFB). According to the signal model, we created an MFB for exponentially decaying sinusoidal signals within the frequency range of the fault case. Based on initial experiments with single-pulse signals, the decay constant τ in the MFB was assumed to be a constant value with 20 ms.

**Figure 12:**
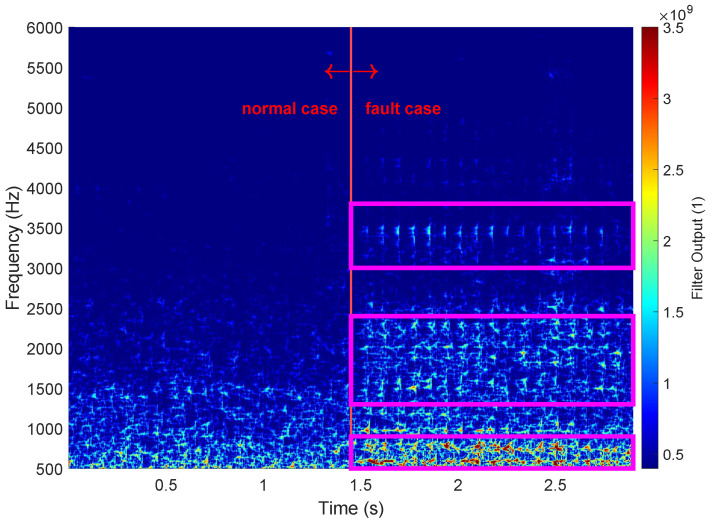
Spectrogram-like filter output generated with an MFB designed on basis of the exponential decaying oscillations. The time constant τ of the filter was held at a constant value for every frequency (τ = 20 ms). The left side of the plot shows the normal operation state condition of the facility, and the right side shows the fault case data. The magenta boxes on the right indicate frequency ranges where, from a visible inspection, the fault signal is more present than in others.

We validated the signal model on several measurements from the facility where we recreated a fault case, as described in Section 2.3. Figure 12 shows one example measurement, where we reproduced a fault event approximately 1.5 s after starting the recording. The figure displays the output of the MFB used to test the assumption of the fault case signal component, with the left side showing the normal operating condition and the right side showing the fault case. The figure clearly indicates that there are components within the signal class of exponential decaying oscillations in the fault signal. This observation supports the notion that the knocking sound manifests itself with components of a decaying sinusoidal signal class. However, the peaks in the graph are somewhat smeared, indicating that the exponential decay differs at different frequencies. Specifically, the longer tail at lower frequencies implies a lower exponential decay and a higher time constant τ. It is worth noting that some peaks can also be seen in the normal case. This is reasonable since conveyed particles also hit the walls of the screen and thus excite some oscillations. However, the amplitudes of these peaks are lower, and the excitation is random in time, unlike the fault case where the excitation has a specific period: the period of screen vibration $T_{m}$. The periodic nature of the fault signal is further supported by the synchronous peaks of the signal at different frequencies (Figure 12). These clearly indicate the excitation of the different frequencies in a synchronous manner. In comparison to disturbing sounds in the facility with similar characteristics, the pattern of the fault case appears periodically as long as the machine is running, which is not the case for disturbing sounds. This clearly distinguishes fault sounds from disturbing sounds.

**Figure 13:**
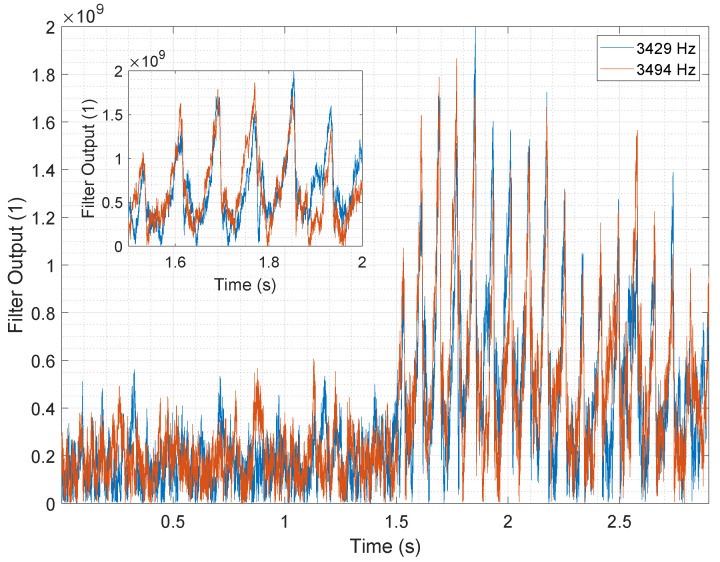
MFB output generated with the matched filter at two specific frequencies (3429 Hz and 3494 Hz).

Variations in the signal parameters such as frequencies and amplitudes of the individual knocking events lead to differences in the individual knocks. Figure 13 clearly shows the differences in the amplitudes of the individual knocking events. The figure shows the MFB output for two different frequencies in the normal state and in the fault case. The data for the fault case (right side of the diagram) show peaks that correspond to the frequency components with exponential decay, as predicted by the signal model. In the normal case (left side of the graph), however, this behavior is not observed.

Figure 13 includes a zoomed-in view of the fault case data, which exhibits a shape that closely resembles the theoretical output of the matched filter. To improve clarity, only the output of two different frequencies is presented, but this pattern can be observed across other frequencies as well. Notably, the peaks of the various frequencies are superimposed, indicating a pulse-like excitation of the different vibration modes. Further analysis using the DFT method showed that the periodicity of the peaks corresponds to the periodicity of the vibrating screen. Overall, the MFB signal investigation demonstrates the validity of the signal model from Equations (6) and (8). Moreover, the analysis revealed that the signal parameters vary across different knocking sounds and that the fault signal is more prominent in certain frequency bands.

In conclusion, we propose a fault signal model with the following characteristics:

- The signal can be modeled with the convolution of a pulse train i[n] and a knocking sound p[n] (Equation (8)): $s_{\mathrm{fault}}\left[n\right]=p\left[n\right]*i\left[n\right]\;\cdot \;u\left[n\right]$.
- The interval of the pulse train corresponds to the vibration period of the screen $T_{m}$.
- The knocking sound p[n] can be modeled as a sum of exponential decaying oscillations:
  $p\left[n\right]=\sum_{k=1}^{N_{f}}A_{k}cos\left(2\pi f_{k}n+\phi_{k}\right)\;\cdot \;exp\left\{-\frac{n}{\tau_{k}}\right\}$.
- The amplitudes, frequencies and phases differ from knocking event to knocking event.
- The exponential decay behaves inversely proportional to the frequency.

## 5. Fault State Detection

Due to the limited availability of actual fault data and to evaluate the suitability of audio features for fault detection at different SNRs, we use artificially generated signals for our analysis. The synthesized signals were generated based on our proposed signal model. Since audio features are the basis of most machine learning based fault detection algorithms, this step is central to many fault detection methods. Rather than exploring a high-dimensional vector space, we compared the individual feature values between fault cases and normal conditions using receiver operating characteristics (ROC) analyses. In this way, we can validate their effectiveness in detecting fault cases, in general. In addition, we will do the same for the GLRT statistics [22] to demonstrate how additional information based on the signal model can be included for subsequent classification purposes.

After evaluating the performance of both approaches on artificially generated data, we then used the recorded audio data from the facility to assess their performance on real datasets as well. These real datasets consist of normal operating sounds, with random interfering noises such as signal horns or maintenance activities present in some recordings, and include the recreated fault data as described in Section 2.3.

**Figure 14:**
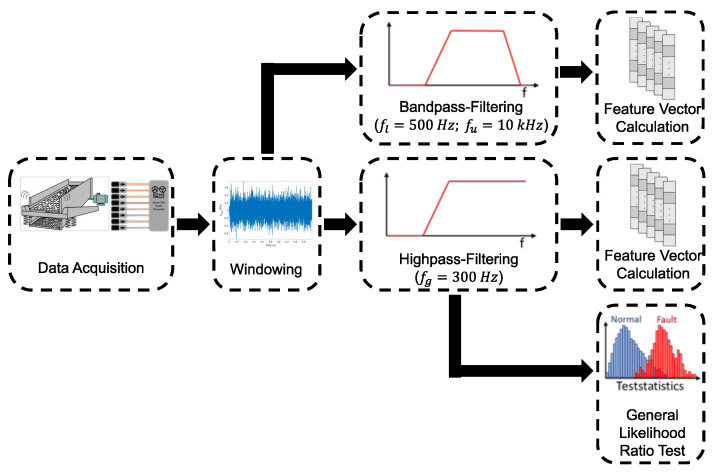
Schematic overview and pre-processing steps for the three detection approaches. The figure illustrates framing, filtering, and feature extraction procedures applied in each method.

Figure 14 gives an overview of the detection approaches discussed and compared in this section. Further, Table 1 summarizes the datasets used to evaluate the different approaches.

Figure 14 shows that the acquired data are first framed for each of the three methods. In the top approach, the data are band-pass-filtered to focus on the frequency band of the fault case before calculating the audio features. In the middle and bottom approaches, the data are high-pass-filtered to only remove the machine component described in Section 3.2, prior to calculating the audio features and for pre-processing in the model-based detection method.

**Figure 15:**
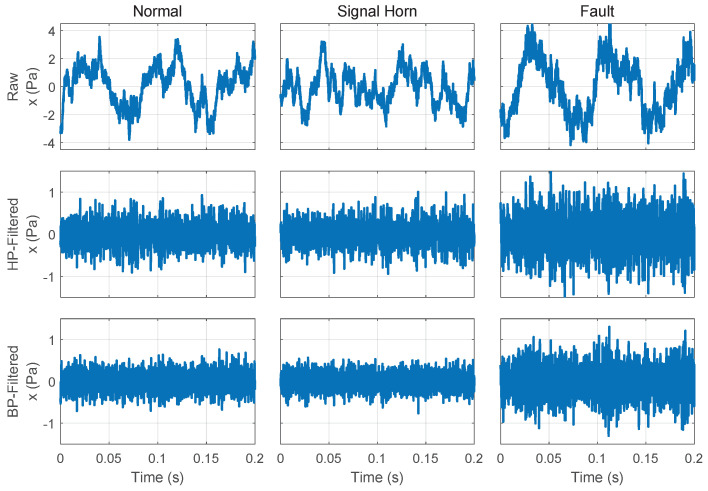
Overview of the different signal pre-processing steps for the normal case (left column), the normal case with an additional signal horn (middle column), and the fault case within the background sound (right column). The second row shows the data after high-pass filtering, where the trend from the vibrating screen was filtered out. The final row presents the band-pass-filtered data, which focuses on the frequency range of the fault case, making the knocking pulses slightly visible.

To demonstrate the effect of the different pre-processing steps, we used real data recorded in the facility. The data include normal operation sounds, normal sounds with an interfering signal horn, and recordings with a fault case. These examples can be seen in Figure 15. This diagram illustrates the effect of filtering out the low-frequency trend caused by the machine sound. In the dataset of the fault case, the knocking signals are barely visible after band-pass filtering due to the low signal-to-noise ratio. However, the knocking signals can be recognized to a certain extent. The data for the normal case is only an example measurement in which the amplitudes are lower than in the fault case. In the normal case, the amplitude fluctuates from measurement to measurement, as the normal operating condition is inherently variable, and can also be higher than the amplitudes in the fault case. The disturbing sound signal cased by a signal horn in the center of the plot is not visible in this time-domain plot.

### 5.1. Audio Feature-Based Fault Detection

We evaluated the detection performance of some selected common audio features from [24], listed in Table 2 over different SNR values. These features were calculated using a Hamming window, as proposed in [24], with a length of 80 ms as recommended in [20].

#### 5.1.1. Artificial Generated Data

We first analyzed the common audio features listed in Table 2 using 60 artificially generated signals with a length of 20 s based on our proposed model. Therefore, we used signals representing both fault cases and normal operating conditions. To replicate the different operational states, the signal power of the material sound was varied within a range identified in our previous investigations. The detection performance is compared using the area under curve (AUC) and the partial area under curve (pAUC) of the ROC analysis, where the pAUC is calculated over a range of false-positive rate (FPR) in the range [0,p] with p=0.1 as suggested in [25].

**Figure 16:**
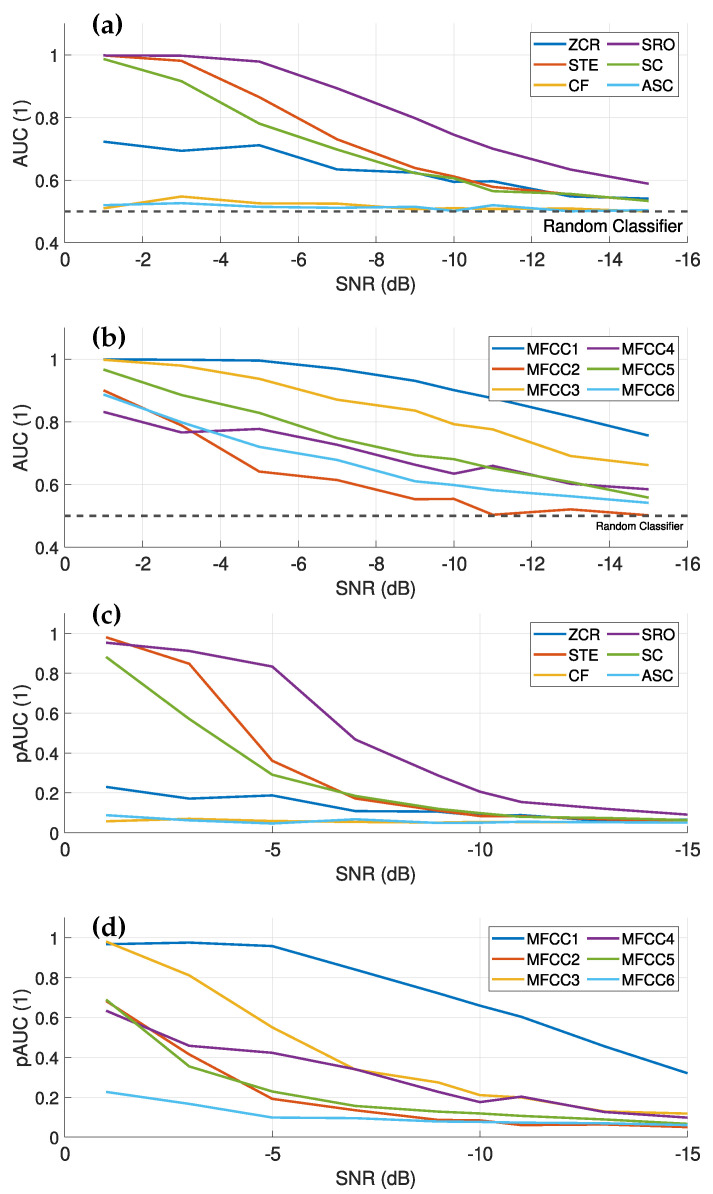
(**a**) the evaluated AUC for different time- and frequency-based audio features over different SNR levels of the generated signals. (**b**) shows the evaluated AUC for the first six MFCC over the SNR levels of the generated signals, where only the first 6 are shown, as higher MFCCs have weaker discriminative properties. (**c**) shows the pAUC of the time- and frequency-based audio features over the SNR values. (**d**) shows the evaluated pAUC for the first six MFCCs over the SNR levels. In (**a**,**c**), a significant decrease in discrimination ability starts at about −5 dB, while this decrease is less steep for the MFCCs. This indicates that the MFCCs have a better discrimination ability at lower SNR values compared to the time- and frequency-based audio features.

According to [26], an AUC value of about 0.9 indicates excellent data discrimination. This is observed for zero crossing rate (ZCR), short time enrgy (STE), spectral roll-off (SRO) and most mel-frequency cepstral coefficient (MFCC)s for SNR values down to about −5 dB, as shown in Figure 16a,b. The evaluation of the different features indicates that the MFCCs are particularly well suited for fault detection, especially at low SNR values. In particular, the first MFCC shows a strong performance, also in terms of pAUC, indicating a good discrimination ability at low-false-positive rates (FPRs). The strong decrease in pAUC for time- and frequency-based features at an SNR of about −5 dB indicates an increase in FPR, which is undesirable for fault detection. In summary, ZCR, STE, SRO and MFCC are effective for fault detection down to an SNR of about −5 dB. However, at lower SNRs, the increasing FPR requires more sophisticated evaluation techniques, such as the inclusion of temporal information. It is noteworthy that the first MFCC does not show such a drastic increase in FPR (decrease in pAUC). Overall, a combination of different features is well suited for machine learning-based recognition algorithms, such as [20], but has the disadvantage of a high false detection rate at an SNR less than −5 dB, as well as in the presence of interfering sounds, both of which are common in real-world applications.

In Section 4, we have shown that the fault signal is characterized by its band-pass nature. Therefore, it is reasonable to apply a band-pass filter to the signals before calculating the features in order to improve the SNR. In a pre-processing step, we therefore filtered the generated signals with a frequency range from 500 to 10,000 Hz. The choice of the lower limit was justified by evaluations of the matched filter bank, as the sound of the material tends to decrease at higher frequencies. Setting this lower limit ensures that the fault case is more prominent compared to the material sound. In addition, the upper limit of 10 kHz for the frequency range was set based on the PSD analysis, which identified this point as the point at which the signal of the fault case is effectively captured.

**Figure 17:**
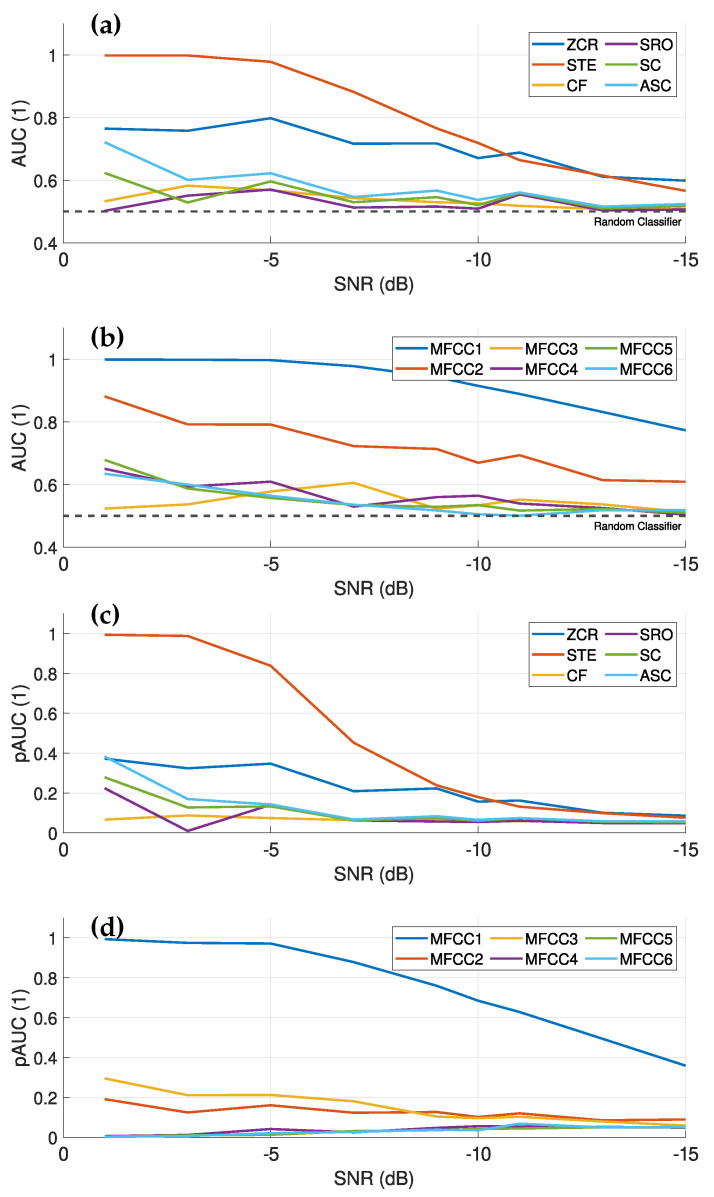
(**a**) Evaluated AUC for different time- and frequency-based audio features over different SNR levels of the band-pass-filtered signals. (**b**) Evaluated AUC for the first six MFCCs over the SNR levels of the band-pass-filtered signals, where only the first six are shown due to weaker discriminative properties of higher MFCCs. (**c**) pAUC of the time- and frequency-based audio features across the SNR values for the band-pass-filtered signals. (**d**) Evaluated pAUC for the first six MFCCs over the SNR levels. The band-pass-filtered signals show that other features now provide the best results, in particular the short time energy (STE). The STE exhibits strong discrimination properties down to an SNR of about −5 dB. The discrimination ability of the first MFCC remains better at lower SNR values, as its decline is more gradual and only starts at an SNR of about −7 dB.

Comparing Figure 16 with Figure 17, it is evident that band-pass filtering considerably improves the discrimination ability of some features, especially in the STE. This improvement is due to the presence of the fault signal in this frequency band, which increases the overall energy level of the signal and thus the STE value. The pAUC at high SNRs (above −5 dB) approaches values close to 1, indicating an excellent separation capability at very low-false-positive rates. In addition, the first MFCC also performs better with band-pass filtering than without.

The ZCR and the second MFCC also exhibit good discrimination capabilities, with stable performance at low SNR values without a drastic drop, making them preferable for applications with an SNR below −5 dB. However, the low pAUC for both indicates a higher-false-positive rate, necessitating more sophisticated classification methods. Nevertheless, the computed audio features from the band-pass-filtered signals show more stable performance at low SNR values than those without filtering, making them also well suited for machine learning-based recognition methods, especially for applications with low SNR values.

#### 5.1.2. Real Data

To summarize, using a combination of different features and pre-processing methods for fault detection with machine learning algorithms seems to be well suited in our application. Since this evaluation is based on artificially generated signals, it provides insights into the performance at different SNR levels but does not take into account random interfering signals that are typically present in real data. Therefore, the evaluation of feature discrimination ability with real data is essential and provides a clearer understanding of performance in the presence of interfering signals. The results for real data are shown in Table 3. For comparison, the table also contains the results of the evaluation of the generated data at an SNR of −7 dB, which corresponds to the average SNR of the real data.

Table 3 illustrates the performance of various features for both generated and real-world data, highlighting notable differences due to the presence of interfering signals in real-world scenarios.

In contrast, in real data, the presence of such interference and the variability of normal operating conditions lead to lower AUC values and lower pAUC values, resulting in higher-false-positive rates compared to the generated data. These discrepancies are due to the additional sounds and dynamic conditions that affect feature performance and reduce discrimination ability. Although the features show promising results with the real-world data, the low pAUC values due to these interferences underline the need for more sophisticated methods to process real-world data. The comparison highlights the importance of refining detection techniques to address these challenges and improve the reliability of fault detection in real-world applications.

Rather than developing interference-resistant machine learning techniques, like presented in [17,18,19,20,27], another effective approach is to use the signal model based on the physical characteristics of the fault, as described in (8). Such an approach focuses directly on the specific features of the fault signal, making it less affected by interference sounds. Since the signal model-based approach targets the particular characteristics of the fault itself, it provides a more reliable detection method that is robust to unwanted interference.

### 5.2. Signal Model-Based Fault Detection

We aim to demonstrate a potential approach for integrating signal model-based information. For this purpose, we employ the GLRT. In statistical terms, the GLRT is a hypothesis testing method. The test statistic is derived from the ratio of the likelihood functions of two hypotheses. In our case, we compare the likelihood function of the null hypothesis (material sound only) with the likelihood function of the alternative hypothesis (material sound plus fault sound). Although our hypotheses do not explicitly address interference signals, this approach is inherently more robust to such additional sounds. This is because it specifically targets the fault signal $s_{\mathrm{fault}}$ within the data, making it less susceptible to the effects of interfering sounds. Thus, the hypotheses are defined as follows:

$H_{0}$: $x\left[n\right]=s_{\mathrm{mat}}\left[n\right]$
$H_{1}$: $x\left[n\right]=s_{\mathrm{mat}}\left[n\right]+s_{\mathrm{fault}}\left[n\right]$

with their corresponding likelihood functions defined in Section 3.3

(9) $$p\left(x|\theta_{0},H_{0}\right)=\frac{1}{\sqrt{det(2\pi \Sigma )}}\;exp\left(-\frac{x^{T}\Sigma^{-1}x}{2}\right)$$

(10) $$p\left(x|\theta_{1},H_{1}\right)=\frac{1}{\sqrt{det(2\pi \Sigma )}}\;exp\left(-\frac{{\left(x-s_{\mathrm{fault}}\right)}^{T}\Sigma^{-1}\left(x-s_{\mathrm{fault}}\right)}{2}\right)$$

The parameter $\theta_{0}$ for the null hypotheses contains the covariance matrix Σ of the material signal and the parameter $\theta_{1}$ contains the covariance matrix Σ of the material signals, the amplitudes, the frequencies, the phases and the exponential decaying constants {A,f,ϕ,τ} of the fault signal model.

The GLRT value is defined as

(11) $$\frac{p(x|\theta_{1},H_{1})}{p(x|\theta_{0},H_{0})}\underset{H_{0}}{\overset{H_{1}}{≷}}\gamma$$

and a decision is made based on the threshold value γ.

Given the unknown model parameters, direct computation of the likelihood functions is not straightforward. As a solution, we propose a procedure involving the parametrization of the signal model, followed by deriving a closed-form solution using maximum likelihood (ML) estimation. Then, we demonstrate a possible method for estimating the model parameters.

Considering only signal frames that contain mainly one pulse (time window of size *T* = 80 ms), we can write the fault model as

(12) $$\begin{array}{ccc} s_{fault}\left[n\right] & \approx & p\left[n\right]=\sum_{k=1}^{N_{f}}A_{k}cos\left(2\pi f_{k}n+\phi_{k}\right)\;\cdot \;exp\left\{-\frac{n}{\tau_{k}}\right\}\\ & = & \sum_{k=1}^{N_{f}}\left(\alpha_{k}cos\left(2\pi f_{k}n\right)+\beta_{k}sin\left(2\pi f_{k}n\right)\right)\;\cdot \;exp\left\{-\frac{n}{\tau_{k}}\right\}\\ & = & H\theta \end{array}$$

with

$$\theta ={\left[\alpha_{1},\beta_{1},\alpha_{2},\beta_{2},\ldots ,\alpha_{N_{f}},\beta_{N_{f}}\right]}^{T}$$

being a $2N_{f}$ dimensional parameter vector and H being a $Nx2N_{f}$ Matrix with elements

$$H_{ij}=\left\{\begin{array}{c} cos\left(2\pi f_{k}n_{i}\right)\;e^{-\frac{n_{i}}{\tau_{k}}}\;\;\;if\;j\;is\;odd\\ sin\left(2\pi f_{k}n_{i}\right)\;e^{-\frac{n_{i}}{\tau_{k}}}\;\;\;if\;j\;is\;even \end{array}\right.$$

and k=round(j/2). Inserting Equations (9), (10) and (12) into Equation (11) gives

$$\frac{exp\left\{-\frac{1}{2}{\left(x-H\theta \right)}^{T}\Sigma^{-1}\left(x-H\theta \right)\right\}}{exp\left\{-\frac{1}{2}x^{T}\Sigma^{-1}x\right\}}>\gamma$$

$$exp\left\{-\frac{1}{2}{\left(x-H\theta \right)}^{T}\Sigma^{-1}\left(x-H\theta \right)-\frac{1}{2}x^{T}\Sigma^{-1}x\right\}>\gamma$$

to simplify the derivation, we take the logarithm of the ratio

$$-\frac{1}{2}\left({\left(x-H\theta \right)}^{T}\Sigma^{-1}\left(x-H\theta \right)-x^{T}\Sigma^{-1}x\right)>ln\left(\gamma \right)$$

(13) $$\frac{1}{2}\left(2\theta^{T}H^{T}\Sigma^{-1}x-\theta^{T}H^{T}\Sigma^{-1}H\theta \right)>ln\left(\gamma \right)$$

Now, for the parameter vector θ, we use the ML solution ${\hat{\theta }}_{ML}$. The ML solution is obtained by maximizing the likelihood function $p(x|\theta_{1},H_{1})$, resulting in

(14) $${\hat{\theta }}_{ML}={\left(H^{T}\Sigma^{-1}H\right)}^{-1}H^{T}\Sigma^{-1}x.$$

Inserting Equation (14) into Equation (13) gives

$$\frac{1}{2}x^{T}\Sigma^{-1}H{\left(H^{T}\Sigma^{-1}H\right)}^{-1}H^{T}\Sigma^{-1}x>ln\left(\gamma \right)$$

(15) $$\frac{1}{2}x^{T}P_{H}x>ln\left(\gamma \right)$$

with

$$P_{H}=\Sigma^{-1}H{\left(H^{T}\Sigma^{-1}H\right)}^{-1}H^{T}\Sigma^{-1}.$$

The matrix *H* remains dependent on the parameters f and τ. These parameters can be estimated via the maxima of a chirp z-transform [28]. The estimated parameters are denoted as $\hat{f}$ and $\hat{\tau }$, respectively. Utilizing these estimations, we can express

$$P_{H}\left(f,\tau \right)\approx P_{H}(\hat{f},\hat{\tau })=\hat{P_{H}}$$

and for the GLRT, it follows that

(16) $$GLRT\approx \frac{1}{2}x^{T}\hat{P_{H}}x>ln\left(\gamma \right)$$

To address the initial assumption in the derivation that only one pulse is present within a single frame, we now provide clarification regarding the data vector x. For the purpose of calculating the test statistic, we consider only a segment of the data vector, corresponding to the defined frame size. This segment is represented as $x_{n_{0}}=\left[\begin{array}{cccc} x_{n_{0}} & x_{n_{0}+1} & \ldots & x_{n_{0}+P-1} \end{array}\right]$, where $P=Tf_{s}$ denotes the duration of the pulse in terms of the number of samples. Thus, the test statistics of the GLRT is written as

(17) $$T\left[n_{0}\right]=\frac{1}{2}x_{n_{0}}^{T}\hat{P_{H}}x_{n_{0}}.$$

The test statistic reaches its maximum when $n_{0}$ corresponds to the starting point of a pulse. Since the starting points of the individual pulses are not known, we calculate the test statistic for each possible $n_{0}$ by sliding a frame of length *P* incrementally over the data. After this process, we determine the maxima of the test statistic within individual frames of length *P* to ensure that the correct values are obtained. As mentioned above, we use the chirp z-transform to estimate the frequency vector $\hat{f}$ and the exponential decay vector $\hat{\tau }$. Based on the results of the fault signal analysis, we limit the frequency range from 300 Hz to 10,000 Hz. In addition, the exponential decay values are limited to the range from 0.01 s to 0.03 s.

#### 5.2.1. Artificial Generated Data

**Figure 18:**
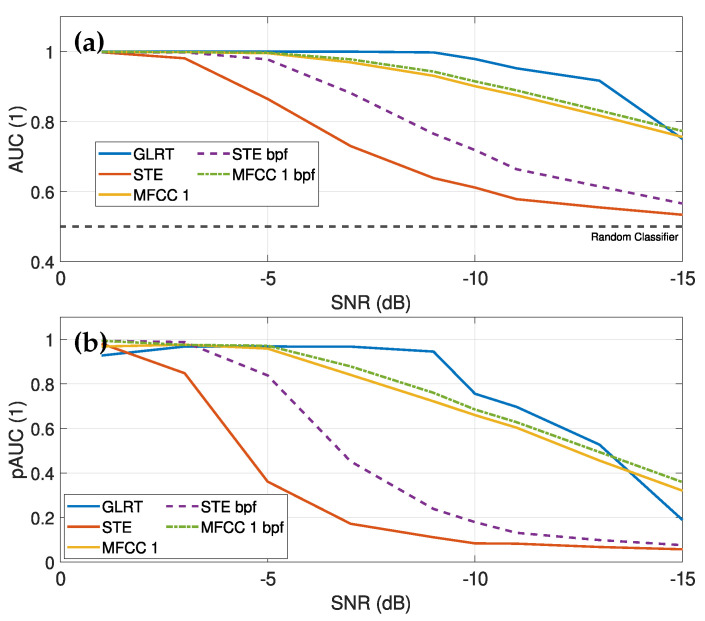
(**a**) Evaluated AUC for the best-performing audio features and the GLRT test statistics over the SNR. (**b**) Corresponding evaluated pAUC value.

To evaluate the detection performance across varying SNR levels, we utilized the same generated data previously used for assessing the audio features. The detection performance results of the GLRT are presented and compared to the detection performances of elected audio features in Figure 18.

The model-based approach (GLRT) demonstrates superior AUC performance compared to all evaluated audio features. However, at an SNR of −13 dB, the AUC declines rapidly and aligns with the performance of the best audio feature, MFCC 1, at an SNR of −15 dB. Throughout the entire evaluated range, the model-based approach consistently outperforms the STE in detection performance. A similar pattern is observed for the pAUC, which shows a sharp decline at −13 dB and falls below the pAUC of MFCC 1 at −15 dB, yet remains significantly better than that of STE. Notably, in the real fault case range of −1.5 dB to −10 dB, the model-based approach surpasses the detection performance of all audio features and is thus suited for the application.

**Figure 19:**
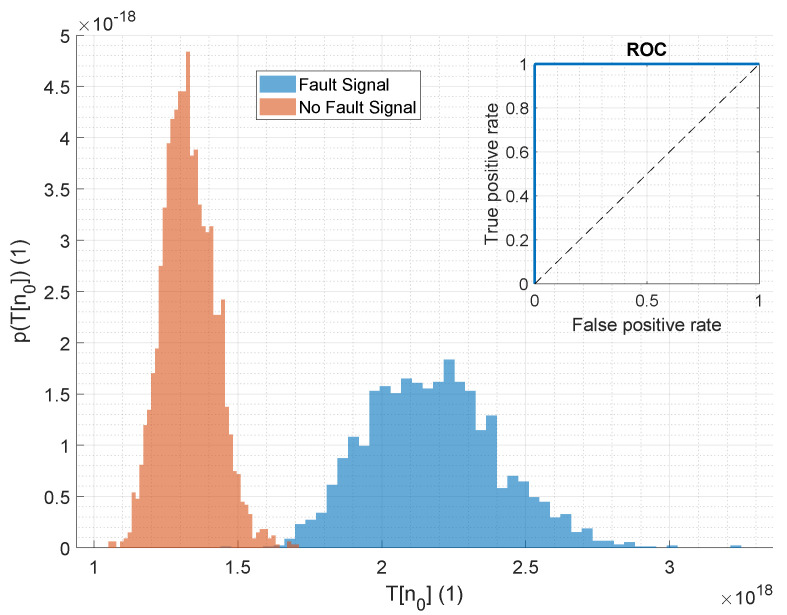
Evaluated histograms of the GLRT values under normal condition (orange) and with an additional fault case (blue) via a Monte Carlo simulation. The test signals for this simulation were generated according to the signal model. A very good separability of the data is given, which can also be recognized from the ROC curve additionally plotted in this figure.

Figure 19 further shows the histogram of the test statistic for the proposed GLRT approach. This result is based on generated data at an SNR of −7 dB, as most fault cases typically have an SNR around this value. It highlights the findings from Figure 18 at an SNR of −7 dB. The histogram demonstrates good separability, and the accompanying ROC curve in the same figure indicates a high level of detection accuracy. This suggests that employing a signal model-based detection approach significantly enhances separability, and the ROC analysis confirms high accuracy in distinguishing the datasets.

#### 5.2.2. Real Data

**Figure 20:**
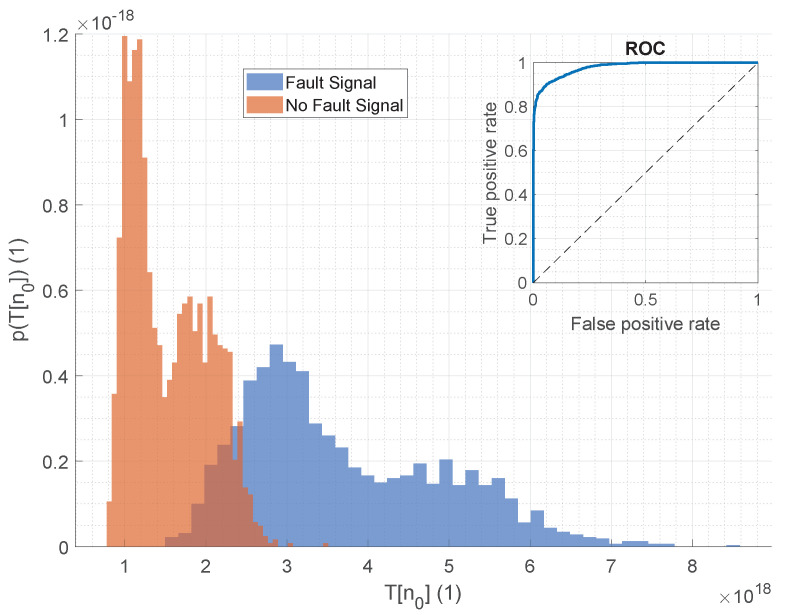
GLRT-statistic for a recreated fault case. Blue: Statistic for the data including the fault signal. Orange: Statistic for the data in the normal operating condition. A clear separability of the data is given, which can also be recognized from the ROC curve additionally plotted in this figure.

However, it should be noted that the generated data were ideally generated according to the signal model, making it an ideal case for the GLRT detector, as it is defined on the signal model. Nevertheless, Figure 20 shows the test statistic for the measured dataset, and there is still good separability with only a small overlap in the histogram.

The overlap in the test statistic distribution is primarily due to a secondary peak near the fault case values, which results from the presence of interfering signals in the real dataset. Unlike the artificially generated data, which consists only of correlated noise, the real-world data includes additional sounds from various sources, such as knocking sounds from machines transporting raw materials (see Figure 12). As the raw material is conveyed along the screen, certain parts of it may strike the machine, causing isolated knocking events. Additionally, signal horns and sounds from workers or maintenance activities contribute to this interference. While these factors lead to the second peak in the distribution, the overall separability remains strong, with the overlap being small. Therefore, despite the background sounds, the signal model-based detection approach continues to maintain good separability between fault and non-fault cases.

Thus, one of the key advantages of the signal model-based detection approach is its robustness to such interfering signals. In contrast, the evaluated audio features are more sensitive to external sounds. In real-world environments, where these disturbances are common, the signal model-based approach consistently outperforms methods based on audio features. This was confirmed by analyzing real-world data, where a decrease in detection performance, particularly in pAUC, was observed for audio features, while the signal model-based approach maintained strong detection performance. A comparison of these results is provided in Table 4.

The GLRT detector is certainly more computationally expensive than simply calculating audio features, primarily due to the need for estimating all the model parameters and computing the matrix $P_{H}$, which involves inverting matrix H. Nevertheless, the computation required for the GLRT approach is still manageable and suitable for the intended application. In this study, our primary focus is to demonstrate how incorporating physically motivated knowledge of the entire system can enhance the detectability of fault cases. We do not address computational costs or algorithm implementation in detail here.

## 6. Conclusions

Based on the proposed signal model and decomposition approach, we have investigated and evaluated the different components of machine sound, material sound and fault sound for huge machines in industrial facilities. Our findings show that the knocking sound of the fault case can be classified as a signal belonging to the class of exponential decaying oscillations. This allows for the development of a fault detection method based on the signal model, which can effectively separate the sound of the normal operation condition and the sound with additional fault sound components.

We have demonstrated that certain standard audio features can be employed for the effective detection of fault cases in our specific application. However, a key limitation of these feature-based approaches lies in their susceptibility to random sound events, which are common in industrial environments. These disturbing sounds, such as those from working personnel or signal horns, can introduce significant signal variability that complicates the fault classification process. This disadvantage is explicitly reflected in the results presented in Table 4, where the real-world dataset exhibits low pAUC values compared to the results of the generated dataset. To mitigate this issue, we applied a band-pass filter as a pre-processing step to enhance the SNR of the fault case, a measure guided by insights from our detailed signal investigation. This pre-processing resulted in improved separability and thus in higher AUC and pAUC values for certain features. For instance, the best-performing audio feature, MFCC # 1, achieved an AUC of 0.85 but still with a pAUC of only 0.39. In general, the low pAUC values in the real data demonstrate a high FPR, highlighting the inherent challenge of distinguishing true faults from random interfering sounds. From the ROC analysis, the performance based on the generated data was significantly better as it does not include interfering sounds.

An essential point to highlight is that our proposed GLRT approach offers enhanced resilience against random disruptions, thanks to its strong foundation on the signal model. This technique is specifically designed to identify exponential decaying oscillations within the signals, which makes it well suited for our purpose. It demonstrates excellent behavior by showing clear separability of data and low FPR at high background sounds and with additional random interfering sounds. This is illustrated in Table 4, where the model-based approach for real data exhibits superior AUC (0.98) and pAUC (0.87) values. A slight decrease in the ROC parameters is also visible with this approach in comparison to the generated data, but the model-based approach demonstrates greater robustness to interfering sounds. However, if interfering sounds such as hammer knockings or similar events are present, the detector may misinterpret them as a fault, since they share similar physical characteristics and adhere to the same signal model. Fortunately, such events are quite rare, which makes the detector’s performance significantly more robust. In addition, these events do not occur in a periodic manner, like true fault cases, a distinction that could be used for further improvements to the model-based approach in the future.

The results show that using audio features without band-pass filtering, reliable detection is limited in the best case (generated data with no interfering sounds) for SNR values lower than −8 dB. Using band-pass filtering, it becomes slightly better, close to −10 dB. In comparison, the model-based approach has good detection performance, as low as −13 dB, which shows that it performs better even with high noise signals. While it is true that further post-processing techniques, such as applying different machine learning approaches to the extracted audio features, can lead to improved results, the ROC analysis presented in [20] still demonstrates that, despite these improvements, these methods perform worse than our signal model-based approach (GLRT). This is because such approaches are inherently designed to detect deviations from the normal state, which often include interfering sound signals rather than exclusively true fault cases.

In conclusion, this work highlights the general benefit of developing fault detection methods based on the underlying physical characteristics of fault signals rather than relying solely on standard audio features. This approach not only provides a more robust solution but also enables the method to be generalized to other industrial applications where similar fault sound properties are present.

## Figures and Tables

**Table 1:** List of data for the evaluation of detection approaches.

Dataset	Artificially Generated Data		Real Data	
**Type**	normal data	fault data	normal data	fault data
**Amount**	1200 s	1200 s per SNR value	1200 s	160 s
**SNR**	-	−15 dB to −1 dB	-	≈−7 dB
**Disturbing Sounds**	not included	not included	included	included

**Table 2:** List of used audio features.

Name	Acronym	Domain
Zero Crossing Rate	ZCR	Time-Domain
Short Time Energy	STE	Time-Domain
Crest Factor	CF	Time-Domain
Spectral Roll-Off Frequency	SRO	Frequency-Domain
Spectral Centroid	SC	Frequency-Domain
Audio Spectral Centroid	ASC	Frequency-Domain
Mel-Frequency Function Coefficients	MFCC	Frequency-Domain

**Table 3:** Performance metrics (AUC and pAUC) for different evaluated features on generated and real-world data. The table includes results for features calculated from raw data and those derived from signals pre-processed with band-pass filtering.

	**AUC**			
	**Generated Data (−7 dB)**		**Real Data**	
**Features**	**No bp-Filtering**	**bp-Filtering**	**No bp-Filtering**	**bp-Filtering**
**ZCR**	0.63	0.72	0.63	0.59
**STE**	0.73	0.88	0.75	0.87
**SRO**	0.89	0.51	0.74	0.73
**MFCC # 1**	0.97	0.98	0.77	0.85
**MFCC # 2**	0.61	0.72	0.78	0.69
**MFCC # 3**	0.87	0.61	0.87	0.76
**MFCC # 4**	0.73	0.53	0.61	0.71
	**pAUC**			
	**Generated Data (−7 dB)**		**Real Data**	
**Features**	**No bp-Filtering**	**bp-Filtering**	**No bp-Filtering**	**bp-Filtering**
**ZCR**	0.11	0.21	0.02	0.14
**STE**	0.17	0.45	0.14	0.42
**SRO**	0.47	0.06	0.40	0.22
**MFCC # 1**	0.84	0.88	0.21	0.39
**MFCC # 2**	0.13	0.12	0.12	0.10
**MFCC # 3**	0.34	0.18	0.55	0.23
**MFCC # 4**	0.34	0.02	0.06	0.12

**Table 4:** ROC analysis for the best-performing audio features and the GLRT approach.

Generated Data (−7dB)			Real Data		
Features	AUC	pAUC	Features	AUC	pAUC
**no bp-filtering** **STE**	0.73	0.17	**no bp-filtering** **STE**	0.75	0.14
**bp-filtering** **STE**	0.88	0.45	**bp-filtering** **STE**	0.87	0.42
**no bp-filtering** **MFCC # 1**	0.97	0.84	**no bp-filtering** **MFCC # 1**	0.77	0.21
**bp-filtering** **MFCC # 1**	0.98	0.88	**bp-filtering** **MFCC # 1**	0.85	0.39
**GLRT** $T[n_{0}]$	1.00	0.98	**GLRT** $T[n_{0}]$	0.98	0.87

## Data Availability

The raw data supporting the conclusions of this article can be made available by the corresponding author (christof.pichler@tugraz.at) upon request.
